# Left Ventricular Molecular Signature in Chronic Aortic Regurgitation

**DOI:** 10.3390/ijms27177950

**Published:** 2026-09-07

**Authors:** Bachar El Oumeiri, Laurence Dewachter, Philippe Van de Borne, Géraldine Hubesch, Pascale Jespers, Constantin Stefanidis, Kathleen Mc Entee, Frédéric Vanden Eynden

**Affiliations:** 1 Department of Cardiac Surgery, H.U.B.—Erasme University Hospital, Route de Lennik 808, 1070 Brussels, Belgium; c.stefanidis@hubruxelles.be (C.S.); f.vandeneynden@hubruxelles.be (F.V.E.); 2 Laboratory of Physiology and Pharmacology, Faculty of Medicine, Université Libre de Bruxelles, 1070 Brussels, Belgium; laurence.dewachter@ulb.be (L.D.); ghubesch@gmail.com (G.H.); pascale.jespers@ulb.be (P.J.); kathleen.mc.entee@ulb.be (K.M.E.); 3 Experimental Laboratory of Intensive Care, Université Libre de Bruxelles, 1050 Brussels, Belgium; 4 Department of Cardiology, H.U.B.—Erasme University Hospital, Route de Lennik 808, 1070 Brussels, Belgium; philippe.vandeborne@hubruxelles.be

**Keywords:** aortic regurgitation, left ventricular decompensation, metabolic remodeling, gene expression, oxidative stress, kallikrein–kinin system, calcium handling

## Abstract

Molecular mechanisms underlying the progression from compensated eccentric hypertrophy to heart failure in chronic aortic regurgitation (AR) remain poorly understood. We investigated associated transcriptional changes induced by chronic volume overload in an experimental model of severe AR. AR was induced in male Wistar rats (n = 10) by retrograde aortic valve perforation and compared with age-matched control rats (n = 8). Sixty days after surgery, cardiac remodeling was evaluated by echocardiography, invasive hemodynamics and myocardial gene-expression profiling using RT-qPCR. Chronic AR induced marked left ventricular dilatation and systolic dysfunction, consistent with decompensated eccentric hypertrophy. These functional alterations were accompanied by coordinated transcriptional changes involving pathways related to apoptosis, oxidative balance, metabolic regulation and calcium handling. AR hearts exhibited an increased *BAX/BCL2* ratio, together with reduced *SOD2* and increased *GPX1* expression, suggesting a transcriptional profile consistent with activation of pro-apoptotic and antioxidant responses. Metabolic remodeling was characterized by decreased *AMPKα1*, *PPARγ* and *GLUT4* expression, together with increased *OLR1* and 15-*LOX*. *KLK10* expression was increased. Reduced SERCA2A expression was observed, consistent with transcriptional alterations involving calcium-handling pathways. Chronic AR is associated with marked structural and functional cardiac remodeling accompanied by coordinated transcriptional alterations across multiple pathways implicated in myocardial dysfunction. This provides potential molecular pathways for further investigation.

## 1. Introduction

Aortic regurgitation is among the more prevalent forms of valvular heart disease, with increasing prevalence in ageing populations [1,2]. Severe chronic aortic regurgitation (AR) imposes a sustained volume overload on the left ventricle (LV), leading to progressive chamber dilatation and eccentric hypertrophy. Initially adaptive, this remodeling preserves cardiac output through preload reserve and LV enlargement, allowing patients to remain asymptomatic for prolonged periods despite ongoing hemodynamic stress [3,4,5,6]. Over time, however, persistent overload promotes adverse remodeling, contractile dysfunction and ultimately heart failure [7].

Although surgical intervention remains the definitive treatment for severe AR, no medical therapy has been shown to consistently prevent LV dilatation, reverse remodeling or improve clinical outcomes. Consequently, current clinical management is largely limited to blood pressure control and clinical surveillance, with heart failure–directed therapy guided by standard guidelines once overt decompensation occurs [8,9]. Even after surgical correction, LV reverse remodeling remains incomplete in a substantial proportion of patients, underscoring the persistence of maladaptive myocardial changes beyond the resolution of volume overload [10]. A better understanding of the mechanisms driving the transition from compensated hypertrophy to LV dysfunction is therefore needed.

Previous experimental studies have identified transcriptional changes associated with early compensated remodeling and advanced volume-overload cardiomyopathy in AR [11,12]. However, the critical intermediate phase during which adaptive remodeling evolves toward decompensation remains insufficiently characterized. Defining the molecular alterations occurring during this transition may reveal novel therapeutic targets capable of delaying or preventing irreversible LV dysfunction.

Accordingly, the present study aimed to characterize structural, functional and molecular remodeling during the transition toward LV decompensation in a rat model of chronic AR. We hypothesized that the transition toward LV decompensation is characterized by coordinated molecular remodeling involving oxidative stress, apoptosis, metabolic reprogramming, inflammatory activation and calcium-handling abnormalities, with a potential contribution of the kallikrein–kinin system.

## 2. Results

As illustrated in Table 1a, AR was confirmed by the presence of a regurgitant jet quantified as severe, with a pressure half-time (PHT) < 200 ms.

### 2.1. LV Decompensation and Eccentric Remodeling

Sixty days after AR induction, animals developed marked LV remodeling characterized by chamber dilatation, eccentric hypertrophy and systolic dysfunction (Table 1a). Fractional shortening (FS) was significantly reduced, and ejection fraction (EF) showed a non-significant trend toward reduction (*p* = 0.08), whereas LV end-systolic diameter (LVESD) and end-diastolic diameter (LVEDD) were markedly increased. Relative wall thickness (RWT) decreased despite preserved septal and posterior wall thickness measurements (Table 1a), indicating progressive eccentric remodeling.

Consistent with chronic volume overload, LV outflow tract (LVOT) diameter, stroke volume (SV) and cardiac output (CO) were significantly increased. LV mass and wall stress indices, including Laplace-derived systolic wall stress, maximal wall stress (σmax) and end-systolic wall stress (σEs), were also elevated, supporting the presence of sustained mechanical overload and maladaptive remodeling. Heart rate and Doppler-derived systolic and diastolic time intervals, including pre-ejection period (PEP), LV ejection time (LVET), systolic time (ST), diastolic time (DT) and RR interval (RR), remained unchanged. Accordingly, PEP/LVET and ST/RR ratios were preserved between baseline and day 60 (Table 1a). Together, these findings demonstrate a transition toward LV decompensation despite the maintenance of forward flow.

To determine whether these changes exceeded those associated with normal growth and aging over the same period, corresponding measurements were obtained in control rats (Table 1b). Over 60 days, control animals showed the expected increase in body weight and a modest increase in LV mass but no significant changes in LV dimensions, systolic function, wall thickness, hemodynamic parameters, stroke volume, or cardiac output. Thus, the marked changes in LV dimensions, FS, RWT, stroke volume, cardiac output and wall stress observed in AR rats were not observed in control animals and are consistent with pathological remodeling associated with chronic AR rather than with time-related physiological growth.

### 2.2. Transcriptional Changes Related to Oxidative Balance and Apoptotic Signaling

**Figure 1:**
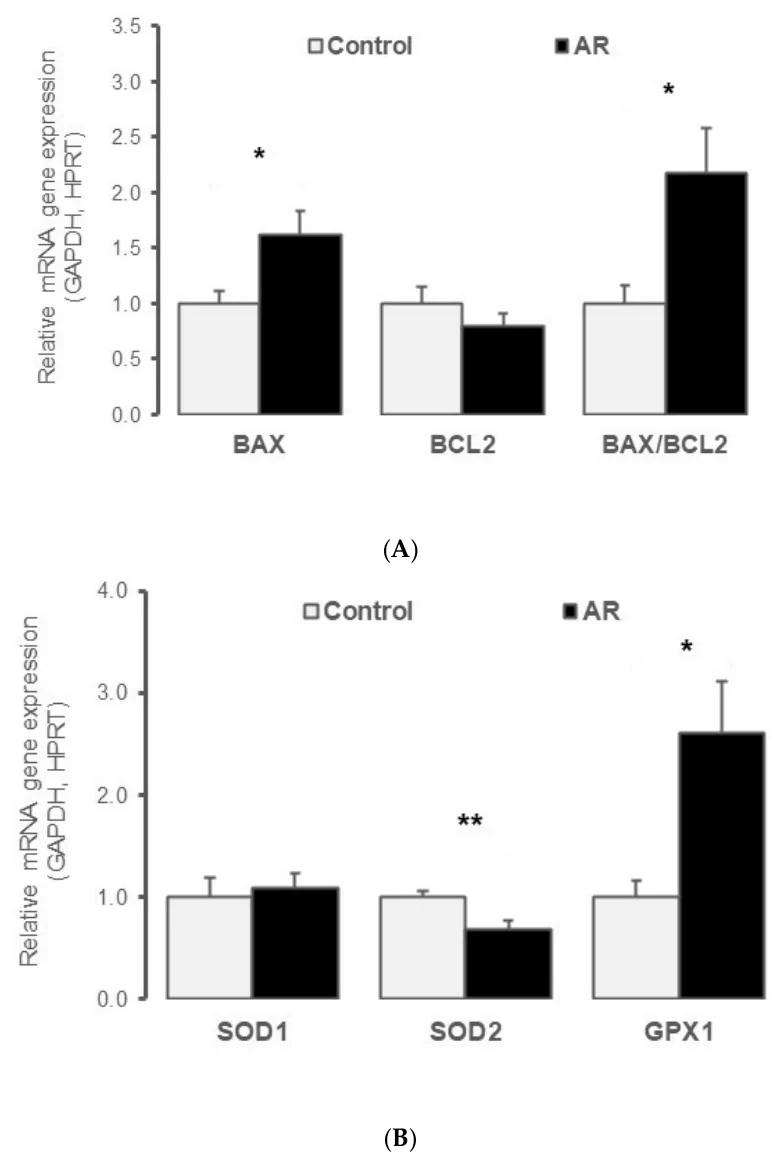
Chronic aortic regurgitation promotes apoptotic signaling and oxidative stress in the left ventricular myocardium. (**A**) Relative mRNA expression of apoptosis-related genes (*BAX* and *BCL2*) and the corresponding *BAX/BCL2* ratio in left ventricular tissue from control (n = 8; white bars) and aortic regurgitation (AR; n = 10; black bars) rats. (**B**) Relative mRNA expression of antioxidant enzymes involved in reactive oxygen species detoxification (*SOD1*, *SOD2*, *GPX1*) in left ventricular tissue from control (n = 8) and AR (n = 10) rats. Data are mean ± SEM; * 0.01 < *p* < 0.05, ** 0.001 < *p* < 0.01 versus controls.

AR was associated with transcriptional changes in genes related to apoptotic signaling and antioxidant defense. The *BAX/BCL2* ratio was significantly increased (Figure 1A), reflecting a transcriptional profile consistent with a shift toward pro-apoptotic signaling. However, confirmation of apoptosis at the protein and cellular levels would require complementary approaches, such as assessment of cleaved caspase-3 or TUNEL staining. This was accompanied by reduced expression of the mitochondrial antioxidant enzyme *SOD2* and compensatory upregulation of *GPX1*, whereas *SOD1* remained unchanged (Figure 1B). The concomitant reduction in SOD2 and increase in GPX1 may reflect an altered transcriptional response to redox imbalance, with GPX1 potentially representing a compensatory response to increased hydrogen peroxide availability. Overall, these findings identify transcriptional alterations in pathways related to redox homeostasis and apoptotic signaling in the LV myocardium of rats with chronic AR but do not by themselves establish functional oxidative stress or apoptosis.

### 2.3. Transcriptional Metabolic Remodeling and Altered Lipid-Related Signaling

**Figure 2:**
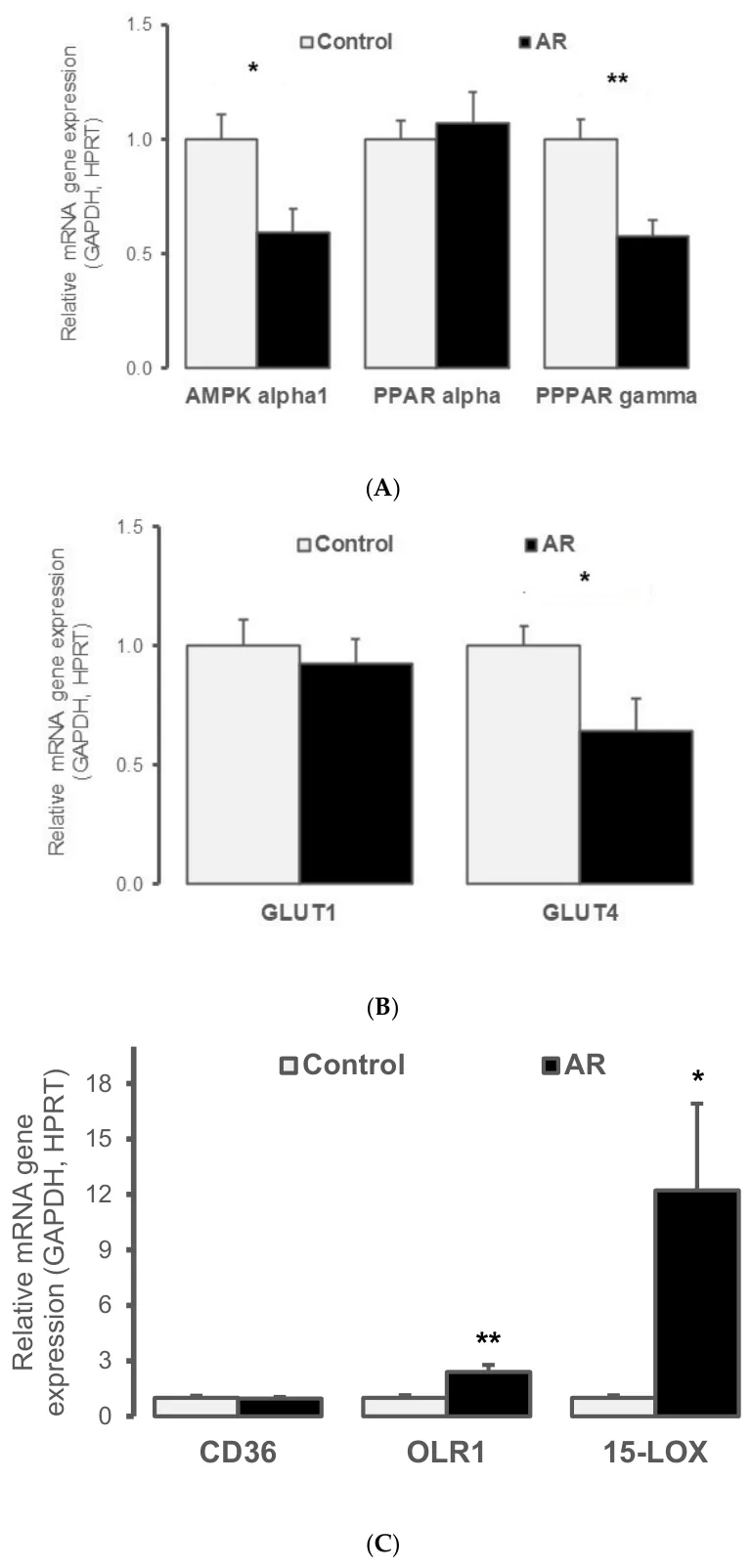
Chronic aortic regurgitation is associated with transcriptional metabolic remodeling and altered lipid-related signaling in the left ventricle. (**A**) Relative mRNA expression of key metabolic regulators (*AMPKα1*, *PPARα* and *PPARγ*) in left ventricular tissue from control (n = 8) and AR (n = 10) rats. (**B**) Relative mRNA expression of glucose transporters (*GLUT1* and *GLUT4*) in left ventricular tissue from control (n = 8) and AR (n = 10) rats. (**C**) Relative mRNA expression of genes involved in oxidized lipid uptake (*CD36*) and lipid peroxidation (*OLR1/1-LOX-1*, 15-*LOX*) in left ventricular tissue from control (n = 8) and AR (n = 10) rats. Data are mean ± SEM; * 0.01 < *p* < 0.05, ** 0.001 < *p* < 0.01 versus control rats.

Chronic AR was associated with coordinated changes in the expression of genes involved in myocardial metabolic regulation. Expression of *AMPKα1* and *PPARγ* was reduced (Figure 2A), together with a significant decrease in the insulin-responsive glucose transporter *GLUT4* (Figure 2B). In parallel, expression of *15-LOX*, encoding 15-lipoxygenase involved in polyunsaturated fatty acid metabolism and lipid peroxidation, and oxidized low-density lipoprotein receptor 1 (*OLR1*; also known as 1-LOX), a scavenger receptor mediating uptake of oxidized lipoproteins, was increased (Figure 2C). In contrast, *CD36* and *PPARα*, which are involved in fatty acid uptake and metabolic regulation, respectively, remained unchanged (Figure 2A,C). These findings indicate transcriptional metabolic remodeling and alterations in lipid-related pathways in the LV myocardium during chronic AR. However, in the absence of protein-level and metabolite measurements, these transcriptional changes cannot be interpreted as evidence of a functional shift in myocardial substrate utilization.

### 2.4. Transcriptional Changes in the Kallikrein–Kinin System

**Figure 3:**
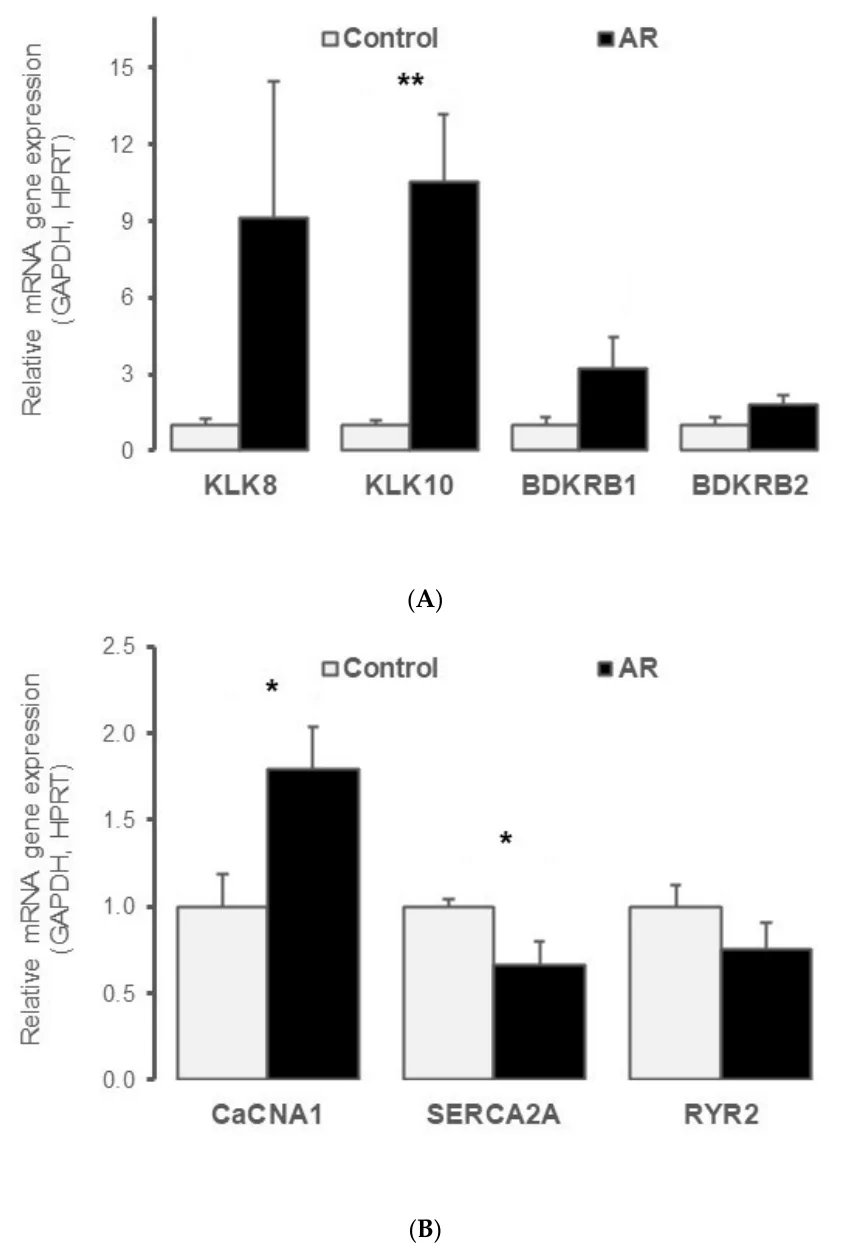
Chronic aortic regurgitation is associated with transcriptional changes in kallikrein–kinin and calcium-handling pathways. (**A**) Relative mRNA expression of kallikrein–kinin system components (*KLK8*, *KLK10*, *BDKRB1* and *BDKRB2*) in left ventricular tissue from control (n = 8) and AR (n = 10) rats. (**B**) Relative mRNA expression of calcium-handling genes (*SERCA2A*, *RYR2* and *CaCNA1*) in left ventricular tissue from control (n = 8) and AR (n = 10) rats. Data are mean ± SEM; * 0.01 < *p* < 0.05, ** 0.001 < *p* < 0.01 versus control rats.

Chronic AR was associated with transcriptional changes involving components of the kallikrein–kinin system (Figure 3A). *KLK10* expression was significantly increased, whereas *KLK8* and bradykinin receptor *BDKRB1* showed non-significant trends toward increased expression (*p* = 0.09 and *p* = 0.06, respectively). Expression of the constitutive bradykinin receptor *BDKRB2* remained unchanged. These findings provide a transcriptional signal suggesting possible involvement of the kallikrein–kinin signaling pathway in the LV myocardium during chronic AR, driven primarily by the significant change in *KLK10*. The non-significant changes in *KLK8* and *BDKRB1* should be interpreted cautiously and require confirmation at the protein and functional levels.

### 2.5. Transcriptional Changes in Calcium-Handling Pathways

As illustrated in Figure 3B, chronic AR was associated with changes in the expression of genes involved in myocardial calcium handling. *SERCA2A*, a key regulator of sarcoplasmic reticulum calcium reuptake, was significantly reduced, whereas expression of the L-type calcium channel subunit (*CaCNA1*) was increased. In contrast, RYR2 expression remained unchanged. This transcriptional profile suggests remodeling of calcium-handling pathways in the LV myocardium during chronic AR and may contribute to the impaired systolic function observed in this model. However, confirmation at the protein and functional levels would be required to establish alterations in calcium handling.

## 3. Discussion

The present study provides an integrated characterization of LV remodeling during the transition from compensated volume overload to ventricular decompensation in experimental chronic AR. Sixty days after AR induction, rats developed marked LV dilatation, eccentric hypertrophy, increased wall stress and impaired systolic function. These functional alterations were accompanied by coordinated changes in LV gene expression involving pathways related to redox homeostasis, apoptotic signaling, metabolic regulation, kallikrein–kinin signaling and calcium handling. Collectively, these findings suggest that ventricular decompensation in chronic AR is associated with a multifaceted myocardial transcriptional remodeling response extending beyond the hemodynamic consequences of volume overload. However, because the molecular analyses were limited to mRNA expression, these findings should be considered hypothesis-generating and require confirmation at the protein and functional levels.

The structural and functional alterations observed in our model are consistent with the natural history of chronic AR. Chronic regurgitant volume overload promotes progressive LV enlargement and eccentric hypertrophy through activation of preload reserve mechanisms that initially preserve forward cardiac output and wall stress distribution [3,4,5]. However, persistent overload may eventually exceed these adaptive mechanisms, resulting in increased wall stress, contractile dysfunction and heart failure [3,5,7]. In the present study, the reduction in FS, together with marked increases in LV end-diastolic and end-systolic diameters and decreased relative wall thickness, was consistent with progression toward decompensated eccentric hypertrophy. EF showed a non-significant trend toward reduction (*p* = 0.08), whereas SV and CO remained elevated, suggesting persistence of compensatory hemodynamic mechanisms despite impaired systolic performance [3,5]. These observations support the concept that sustained mechanical stress contributes to the transition from adaptive remodeling to ventricular decompensation.

One of the main findings of this study is the identification of a myocardial transcriptional profile involving genes related to redox homeostasis and apoptosis signaling. AR hearts exhibited decreased expression of the mitochondrial antioxidant enzyme *SOD2* together with compensatory upregulation of *GPX1*. Reduced SOD2 expression may be consistent with altered mitochondrial antioxidant capacity, whereas the concomitant increase in *GPX1* may represent a compensatory response within the glutathione-dependent antioxidant system. Given that SOD2 catalyzes the dismutation of mitochondrial superoxide to hydrogen peroxide, changes in SOD2 expression may alter the balance between different reactive oxygen species and antioxidant pathways. Increased *GPX1* expression could, therefore, reflect an adaptive response aimed at maintaining hydrogen peroxide detoxification through the glutathione peroxidase pathway. Similar dissociation between mitochondrial and compensatory antioxidant pathways has been described in experimental models of chronic cardiac stress [13,14]. However, because biochemical markers of oxidative stress and protein-level measurements were not available in the present study, these findings should be interpreted as a transcriptional signal consistent with altered redox homeostasis rather than as direct evidence of oxidative stress.

The increased *BAX/BCL2* ratio provides a further transcriptional signal related to pro-apoptotic signaling. Oxidative imbalance and mitochondrial dysfunction have been implicated in cardiomyocyte apoptosis and adverse ventricular remodeling in several experimental settings [13,14,15]. Comparable changes in apoptosis-related genes have also been reported in other forms of LV remodeling, such as the post-myocardial infarction myocardium [16], suggesting that alterations in apoptotic signaling may represent a common feature of adverse ventricular remodeling rather than a mechanism specific to chronic AR. Nevertheless, the present findings do not establish the occurrence of cardiomyocyte apoptosis, as no histological or protein-level markers such as TUNEL or cleaved caspase-3 were available. Further studies will therefore be required to determine whether the observed transcriptional changes translate into increased apoptosis and whether this process contributes causally to LV decompensation.

Metabolic remodeling emerged as another prominent feature of the decompensated phenotype. We observed coordinated downregulation of *AMPKα1* and *PPARγ*, two critical regulators of cellular energy homeostasis, together with significantly decreased expression of *GLUT4*, the principal insulin-responsive glucose transporter in cardiomyocytes. AMPK plays a central role in maintaining myocardial energy balance during conditions of increased workload, whereas altered PPARγ signaling contributes to impaired metabolic flexibility [17,18]. Reduced GLUT4 expression may indicate altered regulation of insulin-responsive glucose transport in the myocardium [19]. In parallel, increased expression of *OLR1* and 15-*LOX* suggests changes in pathways involved in oxidized lipid signaling and lipid peroxidation. Both pathways have been implicated in cardiac inflammation, oxidative stress and ventricular dysfunction [20,21]. The absence of significant changes in *PPARα* and *CD36* further suggests that these transcriptional alterations were selective rather than indicative of a generalized change in all major metabolic pathways. Taken together, these findings support the presence of transcriptional metabolic remodeling in chronic AR. However, they do not establish a functional switch in myocardial substrate utilization, as protein abundance, metabolic fluxes and tissue metabolite levels were not assessed. Further studies will be required to determine whether these transcriptional changes translate into altered myocardial energy metabolism.

An additional finding was a transcriptional signal involving components of the kallikrein–kinin system. *KLK10* expression was significantly increased, whereas *KLK8* and *BDKRB1* showed non-significant trends toward increased expression. Previous studies have implicated the cardiac kallikrein–kinin system in experimental volume-overload states, including pathways related to inflammatory signaling and extracellular matrix remodeling [22]. The inducible receptor BDKRB1 is of particular interest because its expression may increase in response to tissue injury and inflammatory stimuli. However, the present data do not establish activation of the kallikrein–kinin system. Rather, the significant increase in KLK10, together with non-significant trends in KLK8 and BDKRB1, provides a transcriptional signal suggesting possible involvement of this pathway in chronic AR. The functional significance of these changes, as well as their relationship to ventricular remodeling, will require confirmation at the protein and functional levels.

Alterations in calcium-handling gene expression provide another potential link between myocardial transcriptional remodeling and impaired ventricular performance. The reduced expression of *SERCA2A*, a major regulator of sarcoplasmic reticulum Ca^2+^ reuptake, may be consistent with altered regulation of intracellular Ca^2+^ cycling and could contribute to impaired contractile performance [23]. In parallel, increased expression of *CaCNA1* may reflect a compensatory or maladaptive transcriptional response to chronic overload. In contrast, *RYR2* expression remained unchanged. Taken together, the reduced *SERCA2A* and increased *CACNA1C* expression suggest transcriptional remodeling of selected calcium-handling pathways in the LV myocardium during chronic AR. In the context of reduced calcium sequestration efficiency, enhanced calcium influx may promote calcium dysregulation and further impair excitation-contraction coupling [23,24]. However, because our analyses were limited to mRNA expression, these findings should not be interpreted as direct evidence of altered calcium handling. Protein-level measurements and functional assessment of Ca^2+^ transients, channel activity and sarcoplasmic reticulum Ca^2+^ cycling will be required to determine whether these transcriptional alterations translate into functional abnormalities.

Several limitations should be acknowledged. First, the study was conducted exclusively in male rats, precluding assessment of potential sex-dependent differences in myocardial responses to chronic volume overload [25]. Additionally, the approximately 30% mortality observed in the AR group raises the possibility of survivor bias in the 60-day molecular analyses, since only animals surviving the most severe hemodynamic insult were available for gene-expression analysis. Second, molecular analyses were restricted to mRNA expression and therefore do not necessarily reflect protein abundance, enzymatic activity or biological function. This limitation is particularly relevant to the interpretation of the findings related to oxidative stress, apoptosis, metabolism, kallikrein–kinin signaling and calcium handling. Third, biochemical and histological analyses that could have provided direct functional confirmation of oxidative stress and apoptosis were not available in this cohort. Fourth, the relatively small sample size may have limited statistical power to detect more subtle changes in some signaling pathways. In addition, the molecular analyses were exploratory and no formal correction for multiple comparisons was applied, which increases the possibility of false-positive findings. Additionally, although significant baseline-to-day-60 changes were observed in the AR group but not in controls, a formal statistical comparison of the temporal trajectories between groups (e.g., a group-by-time interaction analysis) was not performed. This limitation should be considered when interpreting the extent to which the observed changes exceed physiological, growth-related remodeling. Finally, the observational nature of the study precludes establishing causal relationships between the identified molecular alterations and ventricular dysfunction. Future studies integrating transcriptional, protein, biochemical and functional measurements, together with longitudinal assessment of ventricular remodeling, will be necessary to determine which of these pathways contribute directly to progression from compensated hypertrophy to ventricular decompensation. Advanced functional imaging approaches, including global longitudinal strain, may further help establish links between myocardial remodeling and clinically relevant trajectories toward decompensation [26].

## 4. Materials and Methods

### 4.1. Experimental Model of Chronic Aortic Regurgitation

***Animals and study design.*** All experimental procedures were approved by the Institutional Animal Care and Use Committee of the Faculty of Medicine of the Université Libre de Bruxelles (Brussels, Belgium; protocol approval number 644N) and conducted in accordance with the National Institutes of Health Guide for the Care and Use of Laboratory Animals. Twenty-two adult male Wistar rats (502 ± 27 g; Janvier, Le Genest-Saint-Isle, France) were housed under controlled environmental conditions (12 h light/dark cycle) with free access to standard chow and water. As previously described [27], animals were randomly assigned to either a control group (n = 8) or an aortic regurgitation (AR) group (n = 14). Under 1.5% isoflurane anesthesia, severe AR was induced by retrograde perforation of an aortic valve leaflet. The severity of the regurgitant lesion was confirmed intraoperatively by Doppler echocardiography. Echocardiographic measurements were performed by two investigators blinded to group allocation; molecular (RT-qPCR) analyses were performed using automated instrumentation and standardized protocols, and blinding was therefore not applicable, as these analyses did not involve subjective interpretation by an operator. Animals presenting insufficiently severe aortic regurgitation (PHT > 200 msec) were excluded from the study. The AR group showed an overall mortality of approximately 30% (4 animals), related either to aortic tearing during the valve-leaflet puncture procedure (peri-operative) or to pulmonary edema/hemodynamic decompensation occurring predominantly within the first 3 post-operative days; some animals were euthanized for humane endpoints when they showed persistent overt heart failure despite diuretic treatment. No mortality occurred in the control group. After these exclusions, the final analyzed cohorts comprised n = 8 control and n = 10 AR rats.

***Experimental timeline.*** AR induction or control surgery was performed on day 0. Echocardiographic and invasive hemodynamic measurements were obtained at baseline and repeated 60 days after surgery. At the end of the follow-up period, animals underwent terminal tissue collection for molecular analyses.

### 4.2. Functional and Hemodynamic Assessment

LV structure and function were assessed by transthoracic echocardiography, followed by invasive hemodynamic measurements.

***Echocardiography.*** Transthoracic 2D, M-mode and Doppler echocardiography were performed using a Vivid E90 ultrasound system (GE Healthcare, Wauwatosa, WI, USA) equipped with a 12 MHz phased-array transducer (GE 12S-D). Animals were maintained under 1.5% isoflurane anesthesia throughout the procedure. Continuous electrocardiogram (ECG) monitoring was performed using limb leads. All measurements were obtained by a single experienced operator according to current echocardiographic recommendations [28]. Standard parasternal long-axis, short-axis and apical views were acquired to measure LV dimensions, wall thickness, fractional shortening (FS) and ejection fraction (LVEF). FS was calculated from M-mode tracings using the formula FS (%) = [(LVEDD − LVESD)/LVEDD] × 100, and LVEF was estimated using the Teichholz method. Aortic Doppler recordings were used to determine forward stroke volume (SV) and cardiac output (CO). The severity of AR was quantified by pressure half-time (PHT) of the regurgitant jet, with values < 200 msec indicating severe regurgitation. Relative wall thickness (RWT) was calculated as: RWT = 2 × PWTd/LVEDD, where PWTd represents posterior wall thickness at end-diastole (in mm).

Pre-ejection period (PEP; in msec), LV ejection time (LVET; in msec), RR inter-beat interval (in msec), systolic time (in msec) and diastolic time (in msec) were also derived from simultaneous Doppler and electrocardiographic recordings.

***Invasive hemodynamic measurements.*** Invasive pressure measurements were obtained using a 1.6F micro-tip pressure catheter (Transonic Systems Inc., Ithaca, NY, USA). During the initial surgical procedure, the catheter was introduced through the right carotid artery into the LV to assist AR induction and confirm the severity of the regurgitant lesion, as previously described [27]. Control animals underwent the same procedure without valve perforation. At day 60, invasive hemodynamic assessment was performed in all animals using arterial catheterization before sacrifice. Pressure signals were recorded and analyzed using the ADV500PV acquisition system (Transonic Systems Inc.).

### 4.3. Tissue Collection and Quantitative Gene Expression Analysis

***LV sampling.*** Following completion of the 60-day protocol, animals were euthanized by exsanguination under deep 5% isoflurane anesthesia. Hearts were rapidly excised and the LV was carefully dissected, snap-frozen in liquid nitrogen and stored at –80 °C until further molecular analysis.

***RNA extraction and reverse transcription.*** Total RNA was extracted from snap-frozen LV myocardium using TRIzol reagent (Invitrogen, Merelbeke, Belgium) and purified using the RNeasy^®^ Mini Kit (QIAGEN, Hilden, Germany), according to the manufacturer’s instructions. RNA concentration and purity were assessed spectrophotometrically using a Nanodrop ND-1000^®^ (Isogen Life Sciences, De Meern, The Netherlands), and RNA integrity was verified by agarose gel electrophoresis with GelRed^®^ (Biotium, Hayward, CA, USA) staining. cDNA was synthesized from 1 µg of total RNA using random hexamers and Superscript II Reverse Transcriptase (Invitrogen, Merelbeke, Belgium), following the manufacturer’s protocol.

***Real-time quantitative polymerase chain reaction (RT-qPCR).*** Gene expression was quantified by RT-qPCR using SYBR Green chemistry and gene-specific primers (Table 2). Candidate genes were selected to evaluate pathways involved in apoptosis (*BCL2* and *BAX*), oxidative stress (*GPX1*, *GSR*, *SOD1* and *SOD2*), metabolic regulation (*AMPK*, *PPARα* and *PPARγ*), glucose (*GLUT1* and *GLUT4*) and lipid transport (*CD36*), oxidative injury (*OLR1* and *15-LOX*), kallikrein–kinin signaling (*KLK8*, *KLK10*, *BDKRB1* and *BDKRB2*) and excitation-contraction coupling (*ATP2A2*/*SERCA2*, *RYR2* and *CaCNA1*). Whenever possible, intron-spanning primers were used. Relative gene expression levels were normalized to the housekeeping genes *GAPDH* and *HPRT1* and quantified using the Pfaffl method [29]. All reactions were performed in triplicate, using an iCycler detection system (BioRad Laboratories, Hercules, CA, USA).

### 4.4. Statistical Analysis

Statistical analyses were performed using StatView 5.0 (SAS Institute, Cary, NC, USA). Sample size was based on previous studies from our group using the same experimental model [27] rather than a formal a priori power calculation, which is acknowledged as a limitation.

Data from the tables are presented as mean ± standard deviation (SD), whereas data shown in the figures are presented as mean ± standard error of the mean (SEM). The number of animals analyzed is indicated by n and represents independent biological observations. For the figures, data are presented as mean ± standard error of the mean (SEM). For longitudinal echocardiographic and hemodynamic measurements, baseline and 60-day values were compared using paired Student’s *t*-tests. Normality of the data was assessed using the Shapiro–Wilk test.

For gene expression analyses, comparisons between control and AR groups were performed using an unpaired Student’s *t*-test when the normality assumption was met and the Mann–Whitney test when it was not. Because these molecular analyses were exploratory and hypothesis-generating, no formal correction for multiple comparisons was applied. This approach is acknowledged as a limitation, given the number of genes investigated.

All statistical tests were two-sided, and a *p* value < 0.05 was considered statistically significant. Exact *p* values are reported where appropriate; for graphical presentation, statistical significance is indicated as follows: * 0.01 < *p* < 0.05, ** 0.001 < *p* < 0.01 and *** *p* < 0.001.

## 5. Conclusions

In conclusion, chronic AR is associated with a multifaceted myocardial transcriptional signature involving oxidative imbalance, pro-apoptotic signaling, metabolic remodeling, kallikrein–kinin pathway signaling and altered calcium-handling gene expression. These changes accompany LV structural and functional decompensation and identify molecular pathways potentially involved in myocardial remodeling during chronic volume overload. Further protein-level, biochemical and functional studies are required to establish their mechanistic and therapeutic relevance.

## Figures and Tables

**Table 1:** (**a**) Echocardiographic and hemodynamic evidence of left ventricular decompensation 60 days after induction of chronic aortic regurgitation (AR group). (**b**) Echocardiographic and hemodynamic parameters measured at baseline and 60 days in control rats.

(**a**)			
**Parameter**	**Baseline**	**After 60 Days**	***p***
Body weight, g	502 ± 27	577 ± 24	***
**LV Systolic Function**			
FS, %	42 ± 2	33 ± 1	***
EF, %	76 ± 3	65 ± 4	NS
**LV Structure**			
LVESD, mm	4.6 ± 0.2	7.2 ± 0.3	***
LVEDD, mm	7.9 ± 0.2	10.7 ± 0.3	***
LV Mass, mg	1094 ± 140	1750 ± 209	**
**Wall Thickness**			
SWTs, mm	3.03 ± 0.18	2.85 ± 0.22	NS
SWTd, mm	2.20 ± 0.18	2.38 ± 0.24	NS
PWTs, mm	3.00 ± 0.14	2.89 ± 0.24	NS
PWTd, mm	1.88 ± 0.17	1.76 ± 0.15	NS
RWT	0.49 ± 0.05	0.33 ± 0.03	*
**LVOT/Aortic Outflow**			
LVOT diameter, mm	2.41 ± 0.06	2.82 ± 0.06	***
LVOT VTI, mm	66 ± 4	81 ± 3	**
**Hemodynamics**			
HR, beats/min	315 ± 19	301 ± 15	NS
Systolic BP Pre, mmHg	119 ± 2	115 ± 2	NS
Diastolic BP Pre, mmHg	82 ± 1	59 ± 3	***
Systolic BP Post, mmHg	106 ± 3	/	
Diastolic BP Post, mmHg	63 ± 3	/	
**Time Intervals**			
PEP, msec	28 ± 2	31 ± 3	NS
LVET, msec	81 ± 1	87 ± 3	**
ST, msec	109 ± 3	119 ± 4	**
DT, msec	88 ± 9	85 ± 8	NS
RR, msec	197 ± 11	202 ± 9	NS
PEP/LVET	0.34 ± 0.02	0.36 ± 0.03	NS
ST/RR	0.58 ± 0.04	0.59 ± 0.02	NS
PHT, msec	/	92 ± 7	
**Output Parameters**			
SV, mL	0.30 ± 0.02	0.51 ± 0.04	***
CO, mL/min	96 ± 8	153 ± 13	**
**LV Wall Stress**			
Laplace-s	46 ± 4	76 ± 7	**
Laplace-d	77 ± 6	73 ± 9	NS
σmax	224 ± 18	282 ± 29	*
σEs, dyn/cm^2^	60 ± 5	104 ± 11	**
(**b**)			
**Parameter**	**Baseline**	**After 60 days**	***p***
Body weight, g	477 ± 31	548 ± 37	**
**LV Systolic Function**			
FS, %	36.5 ± 6.7	35.75 ± 6.5	NS
EF, %	70.38 ± 9.46	71.38 ± 9.38	NS
**LV Structure**			
LVESD, mm	5.26 ± 0.52	5.69 ± 0.65	NS
LVEDD, mm	8.65 ± 0.7	8.91 ± 0.48	NS
LV Mass, mg	745 ± 258	992 ± 148	*
**Wall Thickness**			
SWTs, mm	2.42 ± 0.34	2.43 ± 0.45	NS
SWTd, mm	1.61 ± 0.13	1.66 ± 0.22	NS
PWTs, mm	2.52 ± 0.12	2.7 ± 0.2	NS
PWTd, mm	1.71 ± 0.11	1.74 ± 0.21	NS
RWT	0.40 ± 0.04	0.39 ± 0.06	NS
**Hemodynamics**			
HR, beats/min	269 ± 49	261 ± 62	NS
Systolic BP, mmHg	125 ± 15	128 ± 8	NS
Diastolic BP, mmHg	86 ± 14	88 ± 9	NS
**Time Intervals**			
PEP, msec	20 ± 7	22 ± 6	NS
LVET, msec	87 ± 15	85 ± 12	NS
ST, msec	109 ± 17	107 ± 16	NS
DT, msec	121 ± 28	130 ± 36	NS
RR, msec	230 ± 40	236 ± 51	NS
PEP/LVET	0.23 ± 0.08	0.26 ± 0.06	NS
ST/RR	0.47 ± 0.04	0.45 ± 0.05	NS
PHT, msec	/	/	
**Output Parameters**			
SV, mL	0.29 ± 0.06	0.29 ± 0.09	NS
CO, mL/min	79 ± 21	78 ± 30	NS

FS, fractional shortening; EF, ejection fraction; LVESD, left ventricular end-systolic diameter; LVEDD, left ventricular end-diastolic diameter; SWTs/SWTd, septal wall thickness at systole/diastole; PWTs/PWTd, posterior wall thickness at systole/diastole; RWT, relative wall thickness; LVOT, left ventricular outflow tract; VTI, velocity-time integral; HR, heart rate; BP, blood pressure; PEP, pre-ejection period; LVET, LV ejection time; ST, systolic time; DT, diastolic time; RR, RR interval; PHT, pressure half-time; SV, stroke volume; CO, cardiac output; σmax, maximal wall stress; σEs, end-systolic wall stress. Data are mean ± SD. * 0.01 < *p* < 0.05; ** 0.001 < *p* < 0.01; *** *p* < 0.001, 60-day AR versus baseline. NS, not significant.

**Table 2:** Primer sequences used for RT-qPCR analysis.

Gene	Primer Sequence (5′–3′)
Glycerol-3-phosphate dehydrogenase (*GAPDH*)	Sense: 5′-AAGATGGTGAAGGTCGGTGT-3′ Antisense: 5′-ATGAAGGGGTCGTTGATGG-3′
Hypoxanthine guanine phosphoribosyltransferase (*HPRT*)	Sense: 5′-ACAGGCCAGACTTTGTTGGA-3′ Antisense: 5′-ATCCACTTTCGCTGATGACAC-3′
AMP-activated protein kinase (*AMPK*)	Sense: 5′-TTCGGGAAAGTGAAGGTGGG-3′ Antisense: 5′-TCTCTGCGGATTTTCCCGAC-3′
Arachidonate 15-lipoxygenase (*15-LOX*)	Sense: 5′-GCACTCTTCCGTCCATCTTG-3′ Antisense: 5′-GCTTCTCCATTGTTGCTTCCT-3′
ATPase sarcoplasmic/endoplasmic reticulum calcium transporting 2 (*Atp2a2*/*SERCA2*)	Sense: 5′-GCAGGTCAAGAAGCTCAAGG-3′ Antisense: 5′-TCTCTGCGGATTTTCCCGAC-3′
Bcl2-associated X apoptosis regulator (*BAX*)	Sense: 5′-CGTGGTTGCCCTCTTCTACT-3′ Antisense: 5′-TCACGGAGGAAGTCCAGTGT-3′
B-cell lymphoma 2 (*BCL2*)	Sense: 5′-TTTCTCCTGGCTGTCTCTGAA-3′ Antisense: 5′-CATATTTGTTTGGGGCAGGT-3′
Bradykinin receptor B1 (*BDKRB1*)	Sense: 5′-AAGCTACGTGCCTGCTCATC-3′ Antisense: 5′-CGGGGACGACTTTAACAGAG-3′
Bradykinin receptor B2 (*BDKRB2*)	Sense: 5′-GCTGTCGTGGAAGTGGCTAT-3′ Antisense: 5′-AAGGTCCCGTTATGAGCAGA-3′
Calcium voltage-gated channel subunit alpha1C (*CacCNA1*)	Sense: 5′-CCTATTTCCGTGACCTGTGG-3′ Antisense: 5′-GGAGGGACTTGATGGTGTTG-3′
CD36 fatty acid transporter (*CD36*)	Sense: 5′-TTTCTGCTTTCTCATCGCCG-3′ Antisense: 5′-GGATGTGGAACCCATAACTGG-3′
Glutathione peroxidase (*GPX1*)	Sense: 5′-CCGACCCCAAGTACATCATT-3′ Antisense: 5′-AACACCGTCTGGACCTACCA-3′
Kallikrein-related peptidase 8 (*KLK8*)	Sense: 5′-CGGAGACAGATGGGTCCTAA-3′ Antisense: 5′-ATCTCTTGCTCGGGCTCAT-3′
Kallikrein-related peptidase 10 (*KLK10*)	Sense: 5′-GCAGGTCTCCCTCTTCCATA-3′ Antisense: 5′-CAGTGGCTTATTTCTCCAGCA-3′
Oxidized low-density lipoprotein receptor 1 (*Olr1*/1-*LOX*)	Sense: 5′-CATTCACCTCCCCATTTT-3′ Antisense: 5′-GTAAAGAAACGCCCCTGGT-3′
Peroxisome proliferator-activated receptor alpha (*PPARα*)	Sense: 5′-TTAGAGGCGAGCCAAGACTG-3′ Antisense: 5′-CAGAGCACCAATCTGTGATGA-3′
Peroxisome proliferator-activated receptor gamma (*PPARγ*)	Sense: 5′-GCGCTAAATTCATCTTAACTC-3′ Antisense: 5′-CTGTGTCAACCATGGTAATTT-3′
Ryanodine receptor 2 (*RYR2*)	Sense: 5′-GGAACTGACGGAGGAAAGTG-3′ Antisense: 5′-GAGACCAGCATTTGGGTTGT-3′
Solute carrier family 2 member 1 (*Slc2a1*/*GLUT1*)	Sense: 5′-TCTTCGAGAAGGCAGGTGTG-3′ Antisense: 5′-TCCACGACGAACAGCGAC-3′
Solute carrier family 2 member 4 (*Slc2a4*/*GLUT4*)	Sense: 5′-AGGCCGGGACACTATACCC-3′ Antisense: 5′-TCCCCATCTTCAGAGCCGAT-3′
Superoxide dismutase 1 (*SOD1*)	Sense: 5′-GGTCCACGAGAAACAAGATGA-3′ Antisense: 5′-CAATCACACCACAAGCCAAG-3′
Superoxide dismutase 2 (*SOD2*)	Sense: 5′-AAGGAGCAAGGTCGCTTACA-3′ Antisense: 5′-ACACATCAATCCCCAGCAGT-3′

## Data Availability

The original contributions presented in this study are included in the article. Further inquiries can be directed to the corresponding author.
